# Gene Set−Based Integrative Analysis Revealing Two Distinct Functional Regulation Patterns in Four Common Subtypes of Epithelial Ovarian Cancer

**DOI:** 10.3390/ijms17081272

**Published:** 2016-08-05

**Authors:** Chia-Ming Chang, Chi-Mu Chuang, Mong-Lien Wang, Yi-Ping Yang, Jen-Hua Chuang, Ming-Jie Yang, Ming-Shyen Yen, Shih-Hwa Chiou, Cheng-Chang Chang

**Affiliations:** 1 Institute of Oral Biology, National Yang-Ming University, Taipei 112, Taiwan; cm_chang@vghtpe.gov.tw (C.-M.C.); shchiou@vghtpe.gov.tw (S.-H.C.); 2 School of Medicine, National Yang-Ming University, Taipei 112, Taiwan; cmjuang@gmail.com (C.-M.C.); monglien@gmail.com (M.-L.W.); chuangjenhua5@gmail.com (J.-H.C.); mjyang@vghtpe.gov.tw (M.-J.Y.); msyen@vghtpe.gov.tw (M.-S.Y.); 3 Department of Obstetrics and Gynecology, Taipei Veterans General Hospital, Taipei 112, Taiwan; molly0103@gmail.com; 4 Institute of Clinical Medicine, School of Medicine, National Yang−Ming University, Taipei 112, Taiwan; 5 Department of Medical Research, Taipei Veterans General Hospital, Taipei 112, Taiwan; 6 Department & Institute of Pharmacology, National Yang−Ming University, Taipei 112, Taiwan; 7 Department of Obstetrics and Gynecology, Tri-Service General Hospital, National Defense Medical Center, Taipei 114, Taiwan

**Keywords:** epithelial ovarian cancer, function, integrative analysis, gene expression microarray, gene set, machine learning

## Abstract

Clear cell (CCC), endometrioid (EC), mucinous (MC) and high-grade serous carcinoma (SC) are the four most common subtypes of epithelial ovarian carcinoma (EOC). The widely accepted dualistic model of ovarian carcinogenesis divided EOCs into type I and II categories based on the molecular features. However, this hypothesis has not been experimentally demonstrated. We carried out a gene set-based analysis by integrating the microarray gene expression profiles downloaded from the publicly available databases. These quantified biological functions of EOCs were defined by 1454 Gene Ontology (GO) term and 674 Reactome pathway gene sets. The pathogenesis of the four EOC subtypes was investigated by hierarchical clustering and exploratory factor analysis. The patterns of functional regulation among the four subtypes containing 1316 cases could be accurately classified by machine learning. The results revealed that the ERBB and PI3K-related pathways played important roles in the carcinogenesis of CCC, EC and MC; while deregulation of cell cycle was more predominant in SC. The study revealed that two different functional regulation patterns exist among the four EOC subtypes, which were compatible with the type I and II classifications proposed by the dualistic model of ovarian carcinogenesis.

## 1. Introduction

Epithelial ovarian carcinomas (EOC) are composed of a group of heterogeneous subtypes classified by their histology and the degree of epithelial proliferation and invasion. Clear cell (CCC), endometrioid (EC), mucinous (MC) and high-grade serous carcinoma (SC) are four common subtypes of EOC. Within the four subtypes, high-grade SC is the most common type accounting for 70% of EOC, followed by CCC, while MC is relatively rare. However, the carcinogenesis of EOC is still poorly understood. Based on the clinicopathological and molecular features, the dualistic model was proposed and divided EOCs into type I and II categories [1]. The type I EOC, including CCC, EC and MC, usually originating from the mutations of KRAS, BRAF, ERBB2, CTNNB1, PTEN and PIK3CA, is genetically stable and has a relatively indolent behavior [2]. The type II EOC, mainly high-grade SC, displays TP53 mutation in over 80% of the cases, exhibits impaired DNA damage repair and has a more uncontrolled cell differentiation and aggressive behavior. This hypothesis was based on the studies performed in the author’s laboratory and correlated with the clinical, pathologic and molecular features of the disease. However, there is no single study, nor integrative analysis to demonstrate this hypothesis and compare the pathogenesis among the four EOC subtypes. As a result, we conducted a gene set-based analysis integrating the microarray gene expression profiles of the four EOC subtypes from the publicly available database. Gene expression microarray is the primary tool for investigating cancers, the analysis of gene expression profiles usually starts with detecting the differentially expressed genes (DEG) by statistical methods, and then the aberrant Gene Ontology (GO) terms or signaling pathways are inferred from the DEGs. This workflow identifies the most significant disease-related genes, function or processes annotated by GO terms or signaling pathways, however, it will focus only on the significant ones and omit those whose *p* values do not reach statistical significance. In fact, genes or GO terms that did not reach the significance also play a role in the carcinogenesis of EOCs. Besides, only limited functions defined by the GO term or canonical pathways are analyzed; the complete information about the regulation of the functions i.e., functionome in EOC is not provided. To address these limitations, we investigated the pathogenesis of the four subtypes of EOC with microarray gene expression profiles of EOC and their functionomes. The biological function was quantized by converting the gene expression profiles to a gene set regularity (GSR) index computed by modifying the DIRAC algorithm [3], which measured the matching degree of gene expression rankings in a given gene set between two different phenotypes, i.e., EOC and the normal ovarian tissue control in this study. This model utilized the gene set definitions from the GO term [4] and Reactome pathway [5] databases downloaded from the Molecular Signatures Database (MSigDB) [6]. These two gene set definitions collect relatively comprehensive biological functions, processes or signaling pathways. We then utilized them to annotate human functionomes. The GO database contains 1454 gene sets, defining biological functions, process and cellular components; the canonical pathway database contains 1330 curated canonical signaling pathways. In our previous study [7], we demonstrated by the GSR indices a stepwise deterioration of cellular function regularity during SC progression from stage I to stage IV according to International Federation of Gynecology and Obstetrics (FIGO). The pathogenesis of SC centered on cell cycle deregulation accompanied with multi-functional aberrations and interactions. To further explore the pathogenesis and relationship among different subtypes of EOCs, we collected the gene expression datasets of the four common subtypes of EOC and normal ovarian samples from the publicly available databases and converted them into the GSR indices, ranging from 0 to 1 and reflecting the regularities of functions defined by the GO terms or Reactome pathways. Then, the pathogenesis of the four EOC subtypes was investigated and compared with the GSR indices by hierarchical clustering, statistical methods and exploratory factor analysis (EFA).

## 2. Results

### 2.1. DNA Microarray Gene Expression Datasets and Gene Sets

DNA microarray gene expression datasets of the four EOC subtypes were downloaded from the National Center for Biotechnology Information (NCBI) Gene Expression Omnibus (GEO) database. Initially, 1855 potentially eligible microarray gene expression profiles were selected. We filtered out the datasets that resulted in the available common gene number less than 8000 during cross-platform integration. A total of 1452 samples, including 85 CCC, 90 EC, 48 MC, 1093 SC and 136 normal ovarian tissue control samples, were utilized in this study (Table 1). Most of the SC samples were not sub-divided into low- or high-grade SC in the GEO database. However, because high-grade SCs constitute around 90% of all SCs, it was reasonable to assume that the majority of the samples were high-grade SC. These samples data were collected from 38 datasets containing six different DNA microarray platforms without missing data. The 136 normal ovarian tissue gene expression profiles were used as controls for all of the four EOC subtypes. The detailed sample information, including the subtypes, platforms and accession numbers was available in Table S1. The 1454 GO term and 674 Reactome pathway gene set definitions were downloaded from the MSigDB, and the versions were “c5.all.v5.0.symbols.gmt” and “c2.cp.reactome.v5.0.symbols.gmt”, respectively. Because various genes were utilized in different microarray platforms, finally, 1446, 1445, 1446, 1350 GO terms and 669, 669, 669 and 614 Reactome pathways were used in computing the GSR indices for the CCC, EC, MC and SC groups, respectively.

### 2.2. Means and Histograms of the GSR Indices of the Four Subtypes

**Figure 1:**
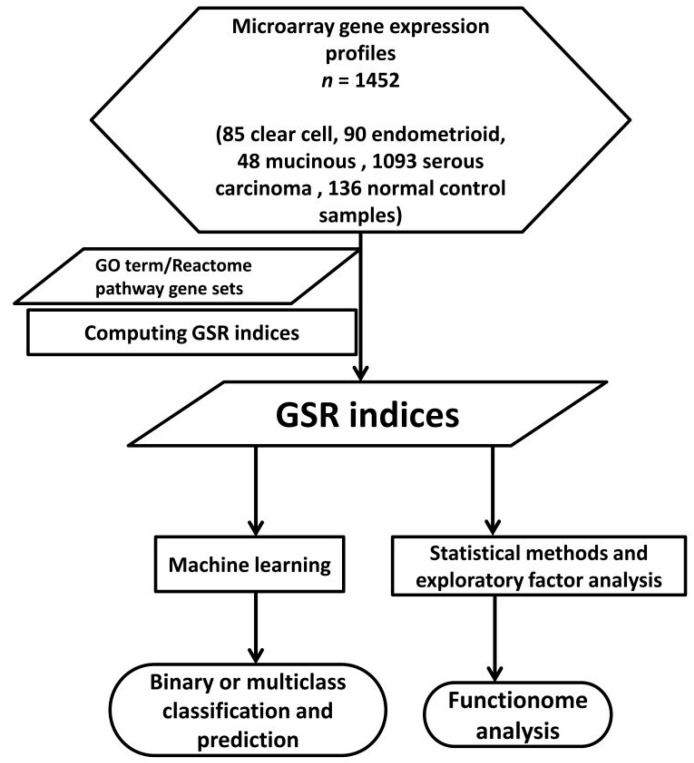
Workflow of the gene set regularity model. The gene set regularity (GSR) index was computed by converting the gene expression rankings of each epithelial ovarian carcinoma (EOC) subtype or normal ovarian control sample through each gene Contrology (GO) term or Reactome pathway gene set. Machine learning algorithm was trained to recognize the patterns consisted of the GSR indices then executed the binary (EOC vs. control) or multiclass (four EOC subtypes + control) classifications. Functionome analyses were carried out by statistical methods, hierarchical clustering and exploratory factor analysis.

The workflow of the GSR model was displayed on Figure 1, and the detailed procedures of computation were described in Methods. The GSR indices ranged from 0 to 1, 0 represented the most chaotic regulation of the function; while 1 represented the functional regulation of the EOC was completely unchanged in comparison with the most common gene expression ranking in the normal control population. The mean of total GSR indices for each subtype group was smaller than the normal control group, and the difference was statistically significant with a *p* value <0.001. The CCC and EC groups had similar means of the GSR indices, and the SC group has the smallest mean of the GSR indices, as listed in Table 1. It indicated the EOC groups exhibited more deregulated functions defined by the GO terms than the normal controls, while the SC group had the worst deregulation state.

**Figure 2:**
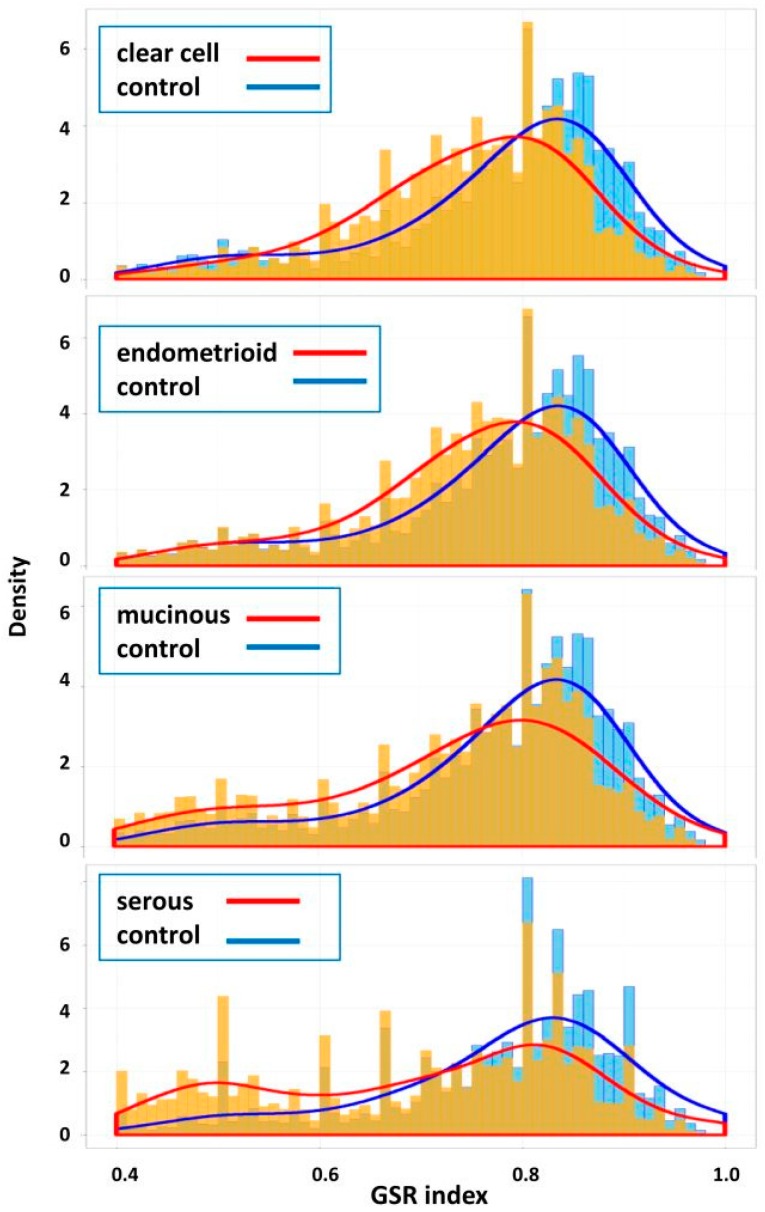
Histograms of the four subtypes. The gene set regularity (GSR) indices for each subtype and normal control group were displayed on the histograms by density. The GSR indices for the two groups showed two distinguishable distributions on the histograms; the distribution consisted of the GSR indices for the EOC subtypes (orange) located on the left side had smaller levels, indicating the biological functions were generally more deregulated in the EOC subtypes than the normal control group (blue).

When displayed on the histograms (Figure 2), two distinguishable distributions of the GSR indices appeared in each EOC subtype; the distribution located on the left side consisted of the GSR indices for the EOC subtype had a smaller levels than the normal control distribution on the right side, indicating the biological functions were generally more deregulated in the EOC subtypes than the normal ovarian tissue controls. Especially, a second peak was observed on the left side of the histogram for the SC group, indicating the existence of a group of more severely deregulated functions in the SC group.

### 2.3. The Relationships of the Four Subtypes

**Figure 3:**
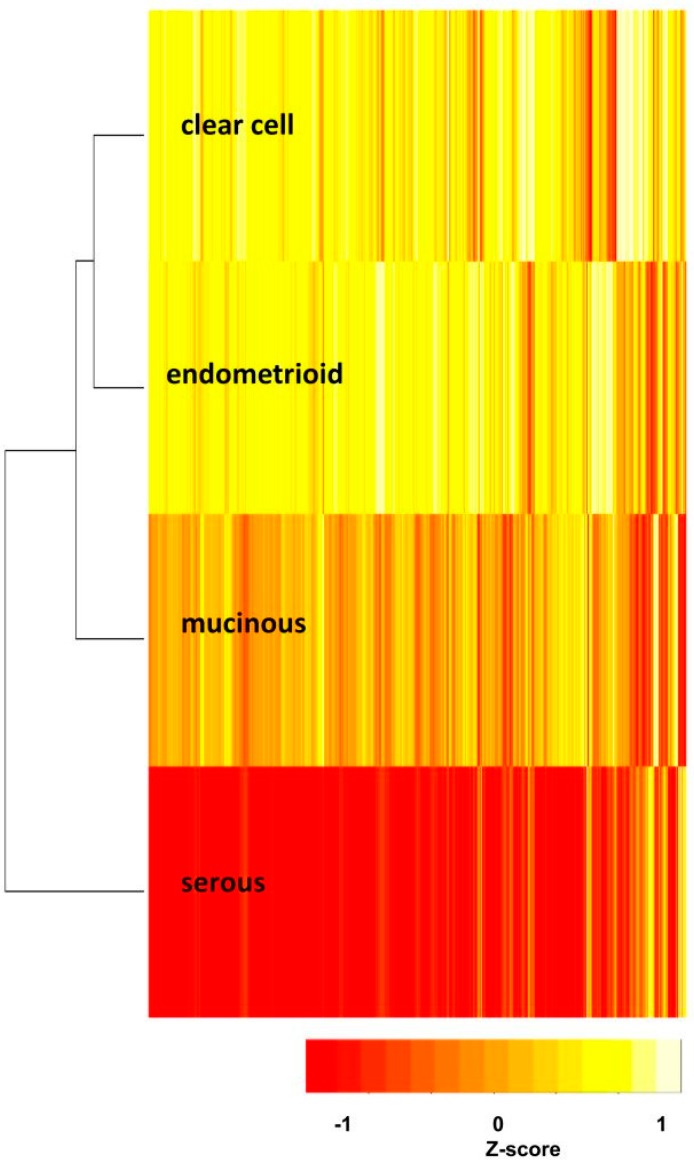
Heatmap and dendrogram of the four subtypes. The heatmap and dendrogram (left side of the heatmap) demonstrated the relationships of the four EOC subtypes. The heatmap showed the CCC and EC groups were the closest, while the SC group exhibited farthest relationship from the others and the most seriously deregulated functions. The red color in the heatmap was correlated with lower, and yellow color with higher value of gene set regularity index.

To discover the relationships of the four EOC subtypes, the GSR indices of each gene set for the four EOC subtypes were averaged then classified by hierarchical clustering and displayed on the heatmap and dendrogram (Figure 3). Grossly, the four EOC subtypes showed distinguishable patterns on the heatmap; the patterns between CCC and EC were more similar, while SC’s pattern was quite distinct from the others and showed the worst regularity of function. Their relationships were also demonstrated by the dendrogram (Figure 3). The CCC and EC groups had the closest relationship, followed by MC, while SC group was the farthest from the other three subtypes.

### 2.4. Functional Regulation Patterns Classified and Predicted by Machine Learning

Machine learning can learn from data by building a model and recognizing patterns to make prediction. We trained support vector machine (SVM) [8], a high performance machine learning algorithm to classify and predict among the four EOC subtypes and the normal control datasets with their functional regulation patterns consisted of the GSR indices. The accuracies were tested by five-fold cross-validation. Of 1316 samples, 1052 samples were used for training, and the remaining 264 samples were used for classification and prediction. Each measurement was measured by the cumulative results of repeating 10 times classifications and predictions. The results were shown in Table 2. The accuracies of binary classification (each EOC subtype vs. control) ranged from 98.18% to 100.00%. The classification between the CCC and normal control groups had the best result. The AUC of the test for each subtype ranged from 0.9805 to 1.0000. The accuracy of multiclass classification among the four subtypes and normal control group was 95.55%. The SVM is a widely used, high-performance machine learning algorithm; this result revealed that the GSR indices could provide sufficient and adequate information for SVM to undergo accurate classification and prediction.

### 2.5. Deregulated GO Terms and Reactome Pathways of the Subtypes

The GSR index is computed based on the extent of ranking change within a gene set defined by the GO terms or Reactome pathways between the case and control group, so the GSR index reflects the regulation of function defined by that gene set and can be utilized to evaluate the function regulation by comparing the difference between the EOC and normal control group. In order to compare the four EOC subtypes and normal controls based on the same standard, the GSR indices of the four subtype and normal control groups were computed after standardization by the baseline gene set template derived from the most common gene expression rankings in the normal ovarian gene expression profiles. The output of this calculation contained approximately 1400 or 670 GSR indices computed through the GO or Reactome pathway gene sets for each case and in each subtype. Table 3 displayed the top 15 deregulated GO terms ranked by the *p* values, and the full content was available in Table S2. The first deregulated GO term was “cofactor transport” for the CC and EC groups, “aldo-keto reductase” for MC and “protein tyrosine kinase activity” for the SC group. There were many recurring gene sets existing among the four subtype groups. For example, “oxidoreductase activity” was found in all of the four subtype groups, while “inositol or phosphatidylinositol phosphatase activity” appeared in the CCC, EC and MC groups. These recurring GO terms represented the commonly deregulated functions among the different EOC subtypes. In addition to oxidoreductase activity and cell adhesion, numerous deregulated GO terms in the SC group were associated with cell cycle, including “spindle”, “negative regulation of cell proliferation” and “double stranded DNA binding”, etc.

The Reactome pathways ranked by the *p* values revealed the first and second significant deregulated pathways in the CCC and EC groups were “downregulation of ERBB2 ERBB3 signaling” and pathways related to PI3K-AKT, respectively (Table 4); the full content is available in Table S3. Obviously, numerous significantly deregulated Reactome pathways were involved in the PI3K-AKT pathway. In the SC group, the first, second and fourth deregulated pathways were associated with G protein. The first deregulated Reactome pathway was “Ca dependent events”; it was a downstream pathway of “G protein mediated events” and “PCL beta mediated events” (4th deregulated Reactome pathway). The second deregulated pathway was “DARPP 32 events”, which was a downstream of G protein coupled receptor (GPCR) signaling pathway and associated with neurotransmitter and steroid signaling. However, their roles in the carcinogenesis of EOC were unknown. Many of the subsequently deregulated pathways in the SC group were associated with cell cycle control, such as “G0 and early G1” and “cyclin A/B1 associated events during G2/M transition pathway”, etc.

### 2.6. The Commonly Deregulated GO Term and Reactome Pathway Gene Sets among the Four Subtypes

**Figure 4:**
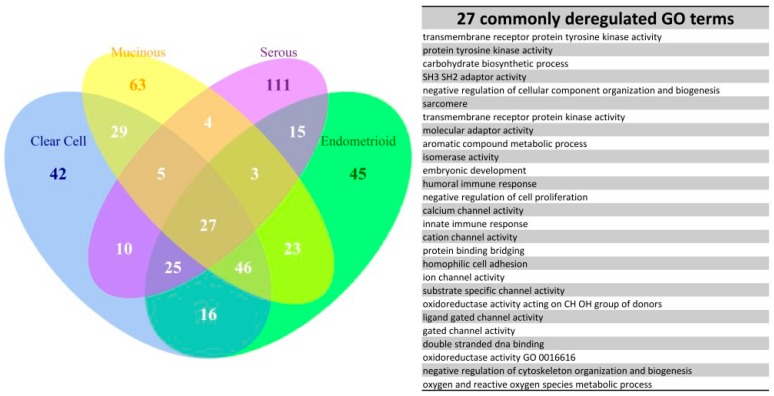
Venn diagram of the top 200 significantly deregulated GO terms for the four subtypes. The results of set analysis for the four ECO subtypes with the top 200 significantly deregulated GO terms ranked by the *p* values were displayed on the Venn diagram to show the gene set numbers of all possible logical relations among the four subtypes. The 27 common deregulated GO terms among the four subtypes were listed on the right side of the diagram.

**Figure 5:**
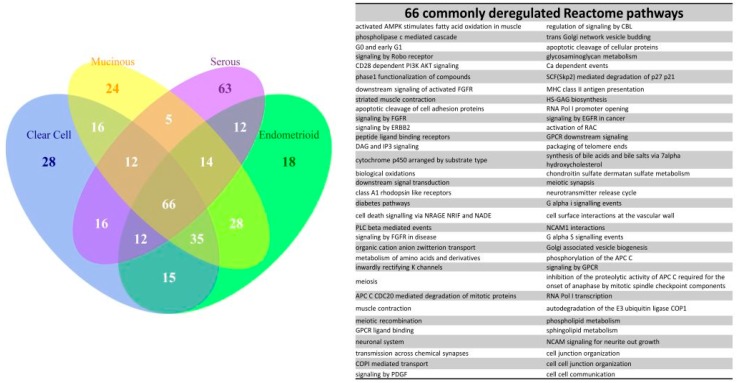
Venn diagram of the top 200 significantly deregulated Reactome pathways for the four subtypes. The results of set analysis for the four EOC subtypes with the top 200 significantly deregulated Reactome pathways ranked by the *p* values were displayed on the Venn diagram to show the gene set numbers of all possible logical relations among the four subtype groups. The 66 common deregulated Reactome pathways among the four subtype groups were listed on the right side of the diagram.

Due to the existence of numerous recurring gene sets among the four subtype groups, we carried out set analysis for the top 200 deregulated GO or Reactome pathway gene sets to find out the similarities of deregulated functions among the four EOC subtypes. The *p* values of those selected gene sets were less than 0.001. The numbers of intersected gene set were displayed on the Venn diagram as shown in Figure 4. There were 27 commonly deregulated GO terms, accounting for 13.5% of all top 200 gene sets among the four subtype groups, including protein tyrosine kinase, cell adhesion, channel activity, oxidoreductase activity, DNA and protein binding etc. The number of common gene sets increased to 73%, or 36.5% of the top 200 gene sets among the CCC, EC and MC groups. Furthermore, the common gene set number was up to 114, or 57% of top 200 gene sets among the CCC and EC groups. It indicated the CCC and EC groups shared more than half of the most deregulated functions and implied a similar pathogenesis between CCC and EC. This finding was compatible with the relationship revealed by the dendrogram on Figure 3. In contrast, the deregulated functions of the SC group were quite different from the other three subtype groups; there were only 39 commonly deregulated gene sets between the MC and SC groups. The set analysis for the Reactome pathway gene sets among the four subtypes showed the number of commonly deregulated Reactome gene sets was 66, it accounted for 33% of the top 200 deregulated pathways. The number of commonly deregulated Reactome gene sets among the CCC, EC and MC groups was 101, or 50.5% of top 200 deregulated gene sets (Figure 5).

### 2.7. The Elements of Carcinogenesis Networks Discovered by Exploratory Factor Analysis

Usually, the pathogenesis of complex diseases, such as EOC, involves a variety of functions” aberrations as well as interactions. EFA is a broadly applied statistical technique to discover the underlying structures, or networks among numerous variables. We carried out the EFA to find out the gene set elements contributing to the EOC carcinogenesis network among 1454 GO terms or 674 Reactome pathways with the gene sets of *p* value <0.0001. The number of “factors”, i.e., structure or network contributing to EOC carcinogenesis, was determined by the function “fa.parallel”. The numbers of factors was 6, 4, 4 and 11 for the CCC, EC, MC and SC groups, respectively. Taking the CCC group as an example, EFA found six networks (factors) of gene sets involved in the carcinogenesis of CCC selected from the deregulated GO terms of *p* value <0.0001; each of the six networks contained 118, 59, 40, 52, 35 and 22 gene set elements, respectively. The 118 deregulated GO terms in the first network were associated with oxidoreductase activity, transmembrane receptor protein tyrosine kinase activity, G protein coupled receptor binding, transcription coactivator activity, chromatin assembly, cell cycle, ion transport, binding and cell adhesion. The second network was composed of the elements associated with sterol binding, cell division, channel activity, oxidoreductase activity, chromatin assembly and inositol/phosphatidylinositol phosphatase activity. They represented two different but overlapped networks of EOC carcinogenesis. The sixth network containing 22 elements was a sub-network of the first one.

Because of the similarity among the CCC, EC and MC groups revealed by the hierarchical clustering and set analysis, we merged the microarray gene expression datasets of the three subtypes (CCC-EC-MC group), recomputed the GSR indices for this group and carried out the EFA to discover the commonly deregulated functions among the three subtypes. The results of EFA showed seven networks of deregulated GO terms. The first network was composed of cell proliferation, oxidoreductase activity, protein binding, cell adhesion, steroid hormone, protein tyrosine kinase activity, GPCR, immune response, GTPase activity and metabolism. The second network was composed of oxidoreductase activity, cell adhesion, extracellular matrix, binding and GTPase activity. The third, fourth and fifth network was associated with channel activity, transport, G protein activity and chromatin assembly, respectively. We also utilized the EFA to analyze the Reactome pathways for the combined CCC-EC-MC group; the results showed the signaling cascades were primarily associated with the PI3K and ERBB pathways. The results of EFA for the SC group showed the deregulated GO terms were predominantly associated with cell cycle, apoptosis, cell proliferation and development. Especially, all of the elements in the 5th network were associated with cell cycle, including “spindle”, “mitotic cell cycle checkpoint”, “M phase of mitotic cell cycle”, “condensed chromosome”, “regulation of mitosis” and “microtubule organizing center”, indicating a series of cell cycle control deregulation. The full EFA results were available in Supplemental Materials (Table S4–S8, for CCC, EC, MC, SC and CCC-EC-MC groups, respectively)

### 2.8. Trees of Deregulated GO Terms for the Four Subtypes

**Figure 6:**
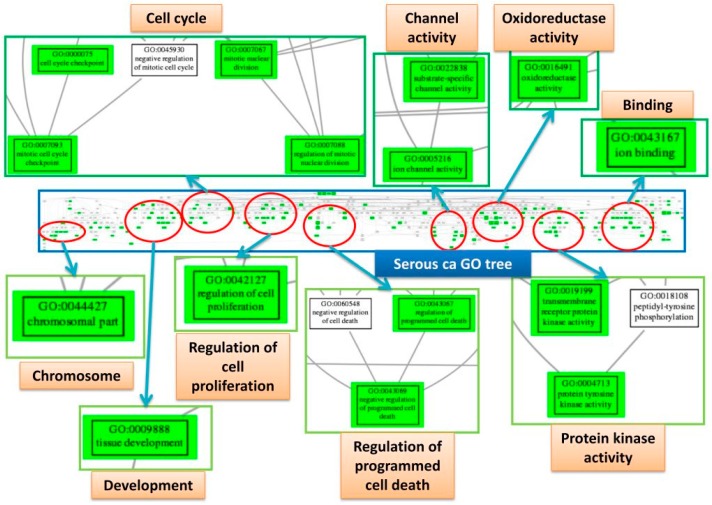
GO tree of SC. This figure displayed the screenshot of the full GO tree for SC (middle). After mapping to the GO tree, the similar GO terms clustered together. Each cluster was circled (red) and some of the important deregulated GO terms (green boxes) in the cluster were magnified to view the details. Each cluster was labeled by the common parental GO term (orange rectangle).

Because the GO terms are structured ontologies established according to their child-parent relationship, the deregulated GO gene set elements from the EFA could be organized and visualized on a directed acyclic graph according to their GO hierarchies. The redundant GO terms could be diminished and simplify the interpretation of EFA results. To establish the tree of deregulated GO terms for each subtype, the deregulated GO gene set elements collected from all factors were merged then remapped to the GO tree by the R package “RamiGO”, which would upload these GO terms to the AmiGO 2 web server for establishment of the GO trees. The deregulated GO tree of SC group is displayed in detail in Figure 6 as an illustration. The full deregulated GO trees of the four subtypes are available in Supplemental Materials (Figures S1–S4). This figure show the screenshot of the full GO tree of the SC group and some important deregulated GO terms. After mapping to the GO tree, the deregulated GO terms with similar functions or properties clustered together and were arranged by their GO hierarchies. Then, the group of clustered GO terms could be summarized by their common parental GO terms. Thus, the deregulated functions, processes or cellular components could be interpreted in a simplified way. Nine groups of clusters could be found in the deregulated GO terms of the SC group, including cell cycle, channel activity, oxidoreductase activity, chromosome, development, regulation of cell proliferation, regulation of programmed cell death and protein kinase activity. The GO tree provided an intuitive way to view the structure of deregulated functions in the carcinogenesis of EOCs. The GO trees of the CCC, EC and MC groups were relatively similar, including components of oxidoreductase activity, cell adhesion, binding, G protein activity, metabolism, channel activity and protein kinase activity. There were overlapping elements among the four EOC subtypes; however, the cell cycle-related GO terms were predominantly observed in the SC group.

### 2.9. Differentially Expressed Genes in the Four Subtypes of EOC

**Figure 7:**
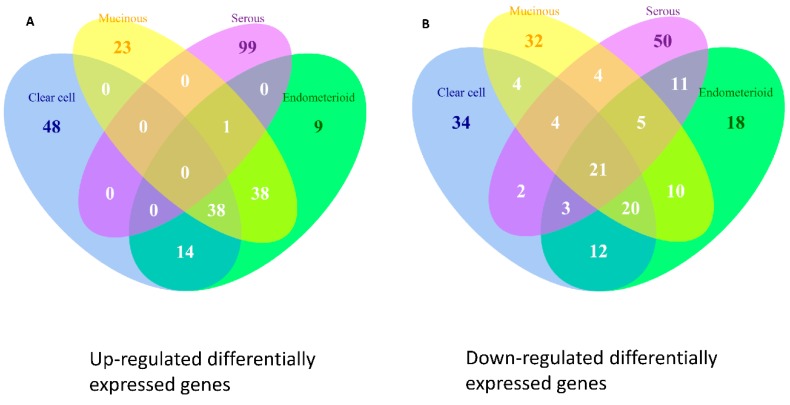
Venn diagram of the top 100 up- and down-regulated differentially expressed genes (DEGs) for the four subtypes. The results of set analysis for the four ECO subtypes with (**A**) the top 100 up-regulated; and (**B**) top 100 down-regulated DEGs were ranked by the *p* values, and the DEG numbers of all possible logical relationships among the four subtypes were shown.

We carried out integrative analysis for microarray gene expression datasets to discover and compare the differentially expressed genes (DEGs) in the four subtypes of EOC. The gene expressions of the samples in each dataset were rescaled to cumulative proportion before integration. Table 5 listed the top 100 down-regulated and up-regulated genes ranked by the *p* values. We found the CCC, EC and MC groups shared many common up-regulated or down-regulated DEGs. We then explored the relationship by set analysis of the top 100 DEGs to find out the similarities on deregulated functions among the four EOC subtypes. The numbers of common GEGs among subtypes were displayed on the Venn diagram (Figure 7). There were 38 commonly up-regulated DEGs, accounting for 38% of all top 100 DEGs among CCC, EC and MC groups; however, no commonly up-regulated DEGs among CCC, EC, MC and SC were found. There were 41% commonly down-regulated DEGs among CCC, EC and MC groups but only 21% among the CCC, EC, MC and SC groups. These findings indicated the distribution of pathogenic DEGs of EOC subtypes was similar among CCC, EC and MC, while SC exhibited a significantly different distribution from the other three subtypes. These results also provided additional evidence supporting the dualistic model of type I and II classifications for ovarian carcinogenesis.

## 3. Discussion

Cancers are usually involved in multiple aberrations of gene and function as well as their interactions. In order to take these features into consideration, we utilized the GSR model to investigate the function regularities in cancers. Instead of detecting the DEGs, the model starts with converting the microarray gene expression profiles into quantized biological functions through a list of gene sets defined by the GO terms or Reactome pathways, and then the pathogenesis is evaluated by comparing the differences of functional regulation between the cases with the normal control groups. These quantized regularities of functions, i.e., the GSR indices, are computed by the modified DIRAC algorithm, which converts the gene expression levels to a gene expression ranking list in a gene set, and then measures the matching degree of gene expression rankings between two different phenotypes. We utilized a baseline gene set expression ranking template, defined as the most common gene expression ranking in the normal control populations for each gene set, as a standard to measure the regularity of gene ranking in either EOC or normal ovarian control sample. Then, the GSR index is computed by measuring the matching degree between the gene expression rankings of each ovarian cancer or normal ovarian control sample with the baseline gene set expression ranking template for each gene set. After being standardized by the baseline gene set template, the GSR indices of the four EOC subtypes can be compared based on the same standard. Besides, the GSR indices are computed based on the gene expression rankings; the gene expression levels are converted into ordinal data, and the ordinal data will encounter less cross-platform bias than the gene expression levels during integrating the datasets from different DNA microarray platforms. Computing the gene expression ranking in a gene set will take the gene interactions in a gene set into consideration. In contrast to the “genome” analyzed with gene expression microarray, this model investigates “functionome” with the GSR indices. By converting tens of thousands of gene expression profiles to approximately one thousand GSR indices, this approach will diminish the data noise, simplify the complexity of the subsequent analyses, and facilitate the performance of machine leaning. Besides, each GSR index is normalized to a value ranging from 0 to 1, in favor of the subsequent analyses.

The functionome of each subtype was computed through either GO term or Reactome pathway gene set database, both databases collect relative comprehensive human biological functions and processes, and provide the browsers for viewing the hierarchy of GO terms (AmiGO 2) [9] and pathways (Reactome Pathway Browser) [10], facilitating the clarification of the relationships among numerous deregulated GO terms or pathways. The functionome was composed of approximately 1400 GO or 600 Reactome GSR indices for each case, when displayed on the heatmap, the functionomes of the four EOC subtypes could be visualized and show distinguishable patterns. These patterns could be recognized, classified and predicted by the machine learning. Our result revealed excellent binary or multiclass classification; it implied that the functionomes composed of GSR indices could be utilized as the basis of molecular classification by machine learning. Subsequently, the pathogenesis of the four subtypes was investigated by evaluating the GSR indexes. From the results of histograms and hierarchical clustering among the four subtypes, it could be found that CCC and EC had the closest relationship, followed by MC, and SC was relatively different from the others in terms of functional regulations. Indeed, the four subtypes shared quite a number of common deregulated functions, including cell adhesion, oxidoreductase activity, protein binding, channel activity and metabolism. However, deregulations of chromatin assembly, ERBB, PI3K-AKT pathways were more common among CCC, EC and MC but not in SC. In contrast, the predominant deregulated functions in SC were cell cycle control.

We further explored the pathogenesis and the relationship among the four subtypes by the EFA. The results of EFA using GO terms disclosed that CCC, EC and MC shared a similar structure of pathogenesis, associated with binding, channel activity, cell adhesion, oxidoreductase activity, protein kinase activity, G protein activity and chromatin assembly. The results of EFA using Reactome pathway gene sets revealed the common deregulation of the PI3K-AKT and ERBB pathways. In contrast, the results of EFA for the SC group revealed the pathogenesis mainly involved in apoptosis, mitosis and cell division and cell cycle checkpoint. Overlapped deregulated functions among the four EOC subtype groups were also found, such as protein tyrosine kinase activity, carbohydrate biosynthetic process, immune response, channel activity, cell adhesion and oxidoreductase activity. The channel activity was demonstrated to be involved in the cell cycle control in the carcinogenesis of EOC [11], and cell adhesion played an important role in the metastasis of EOC [12]. These findings draw the conclusion that the two overlapped, but distinguishable function regulation patterns existing among the four subtypes of EOC. The first pattern observed in the CCC, EC and MC groups had moderate, deregulated functions involved in oxidoreductase activity, channel activity, binding activity, metabolism, chromatin assembly, cell adhesion, PI3K-AKT and ERBB signaling pathway. The secondary pattern, observed in the SC groups, had more severe functional regularity and was predominantly involved in the cell cycle deregulation. These two function regulation patterns were compatible with the type I and type II classifications proposed by the dualistic model of ovarian carcinogenesis: the type I EOCs, including CCC, EC and MC, usually originated from the mutation of KRAS, BRAF, ERBB2, PTEN and PIK3CA, are genetically stable and have a relatively indolent behavior; the type II EOCs, mainly high-grade SC, primarily exhibit a TP53 signature, have a more uncontrolled cell cycle and aggressive behavior. The type I and II EOCs were compatible with the first and second patterns of function regulation in our study, respectively.

This study also showed evidence disclosing the relationship between deregulated functions and carcinogenesis. The association of CCC and EC with endometriosis has been repeated reported [13,14]. The cells in the endometriosis foci will be exposed to the reactive oxygen species (ROS) and are subjected to more DNA damage [15]. As the dendrogram showed in this study, the CCC and EC groups exhibited a relatively close relationship and shared many commonly deregulated GO terms, such as oxidoreductase activity and cell adhesion; both are the characteristic features of the pathogenesis of endometriosis. These findings provided the evidence supporting the role of endometriosis during the carcinogenesis of CCC and EC.

Our results showed the PI3K-AKT signaling pathway was a key element of the pathogenesis of EOCs. PI3K-AKT has been demonstrated to play an important role in the carcinogenesis of EOC, especially in CCC and EC. The deregulation of this signaling pathway may be originated from the loss of PTEN in 40% cases [16], PIK3CA mutation in 33% cases [17] or AKT amplification in 14% cases [18] of CCC patients. PI3K is the major downstream effector of receptor tyrosine kinases (RTK) and GPCR. If PI3K is activated, apoptosis will be inhibited and leads to cell proliferation [19]. Both of PI3K-AKT and G protein deregulation were detected with statistical significances in this study. As the results of CCC-EC-MC combined analysis listed in the Table S9, the GO terms “inositol or phosphatidylinositol phosphatase activity” and “transmembrane receptor protein tyrosine kinase activity” were the first and sixth top deregulated GO gene sets. ERBB2 was the first deregulated pathways for CCC and EC, its expression in EOC varies widely, ranging from 20% to 30% of cases [20]. ERBB is a member of the epidermal growth factor receptor (EGFR) family, it can activate the PI3K-AKT pathway and may represent a prognostic factor in primary EOC [21]. The 9th deregulated Reactome pathway “PI3K events in ERBB2 signaling” in the CCC-EC-MC combined group indicated the interaction between the two important deregulated Reactome pathways in the carcinogenesis of EOC (Table S10).

However, there are limitations when applying the GSR model to investigate the carcinogenesis of EOCs. As an illustration, the TP53 mutation is a common aberration in high-grade SC. The gene set related to TP53 could be found in the list of Reactome pathway database; however, they did not appear on the top of the significantly deregulated pathway list in this study; the first one appearing on the list was the 122th gene set “P53 dependent G1 DNA damage response” with a *p* value of 4.02 × 10^−17^. This finding illustrates the first limitation of this model: if the level of gene expression change does not reach the required extent, the gene expression ranking as well as the GSR index will remain unchanged and the aberration could not be detected. The second limitation is the incompleteness of gene set definitions. For example, there was no definition of PTEN gene set in the GO and Reactome gene set database, so no PTEN aberration was found in this study, although this model discovered a lot of PI3K related functions and pathway aberrations because the PI3K were the effector of PTEN. The third limitation is the false positivity. The third most deregulated Reactome pathway in the MC group was “olfactory signaling pathway” with a *p* value of 1.32 × 10^−12^, which should be independent of the carcinogenesis of MC. This situation can be checked and clarified via the Reactome Pathway Browser. When mapping to the browser, the hierarchy showed the “olfactory signaling pathway” was a member of the GPCR signaling pathway and contained elements involved with the regulation of G protein, and G protein was shown to play an important role in the carcinogenesis of EOC in this study. This false positivity happened because of the presence of the G protein-related gene elements in the gene set. Another limitation of this study was that the DEGs derived from the integrative analysis had not been validated. One of the best ways to validate these DEGs is RNA seq or protein expression for the samples of the four EOC subtypes. We attempted to validate the DEGs in our study by collecting the RNA seq datasets for the four EOC subtypes from two important publically available databases: The Cancer Genome Atlas (TCGA) and NCBI Sequence Read Archive (SRA). However, this validation was not feasible because the available samples of CCC, EC and MC were not enough to get significant statistical significance. Further investigation is still needed for validation of these DEGs.

## 4. Materials and Methods

### 4.1. Computing GSR Indices by Modified Differential Rank Conservation Algorithm

The algorithm of computing the GSR indices was modified from the Differential Rank Conservation (DIRAC). DIRAC is designed to measure the perturbation of a gene set by converting gene expression levels to gene expression rankings, and quantifying the regularity of gene expression ranking in the gene set by computing the ranking matching score, which is a measurement of the degree of each sample’s gene expression ranking of each gene set matching the corresponding gene set ranking template. Instead of measuring the perturbation of gene expression ranking, the GSR index measures the extent of gene expression ranking change between two phenotypes in a gene set, i.e., EOC and normal controls in this study. For this purpose, the GSR indices for both EOC and the normal control are computed by comparing the sample’s gene expression ranking with a standard template derived from the most common gene expression ranking in a gene set among the entire normal ovarian tissue control samples. Then, the EOC pathogenesis was investigated by comparing the EOC and normal control GSR indices. The baseline gene set ranking template was defined as a template of the most common gene ranking among the unaffected controls in a gene set; it is used as a standard template for a gene set from the unaffected population. The baseline gene set raking template for each gene set is established by pairwise comparison between the expression levels of two genes for all possible combinations of gene pair. A gene set contains *m* gene G = {*G*_1_, *G_m_*}, and the corresponding gene expression profile E = {*E*_1_, *E_m_*}, *E_i_* denotes the expression level of gene *G_i_*. Each sample is labeled by a phenotype of case (EOC) and unaffected control group, respectively. The baseline gene set raking template for each gene set is established by pairwise comparison between the expression levels of two genes for all possible combinations of gene pair. The baseline gene rank template B for a given gene set G is the binary vector composed of “A” or “B”, where each component is either “A” if the probabilities Pr(*E_i_* < *E_j_* | phenotype = control) >0.5 or “B” if Pr(*E_i_* < *E_j_* | phenotype = control) ≤0.5. For the expression profile of a given sample *e_n_*, the GSR index for a given gene set is the fraction of the *m* × (*m* − 1)/2 pairs for which the observed gene expression ranking within *e_n_* matches the baseline gene ranking template B. Establishment of the baseline gene set expression ranking template and measurement of GSR indices were executed in R environment, the code and the test datasets are available on the GitHub (https://github.com/carlzang/GSR-model.git).

### 4.2. Microarray Datasets Gene Set Definition and Data Processing

Gene expression microarray datasets were downloaded in a SOFT format after comprehensively searching for all of the available microarray gene expression profiles in the NCBI GEO database. Ovarian carcinoma and normal ovarian tissue control datasets were selected only when the samples originated from the ovarian tissue and definite diagnosis was provided. The gene expression profile was discarded if containing missing data. The manipulation of genes and the corresponding gene expression data in each dataset was based on the HUGO Gene Nomenclature Committee (HGNC) gene symbols approved in 2013. The microarray gene expression datasets were utilized only if the corresponding gene symbol information was provided in the annotation table. The common genes and the corresponding gene expression profiles among all datasets were used in this study. The dataset were discarded if the number of the common genes became less than 8000 during intersecting with other datasets. The gene sets were discarded if the number of gene elements in the gene set is less the 3.

### 4.3. Statistical Analysis

The differences between the four EOC subtypes and the control GSR indices were tested by Mann Whitney *U* test and corrected by multiple hypotheses using false discovery rate (Benjamini-Hochberg procedure). The significance level was set at <0.001.

### 4.4. Classification and Prediction by Machine Learning

GSR index matrices computed through GO term and Reactome pathway gene sets were classified and predicted by the support vector machine (SVM) with kernlab [22], which is an R package for kernel-based machine learning methods and is used to classify patterns of the GSR indices with the setting of kernel = “rbfdot” (Radial Basis kernel “Gaussian”), type = “C−svc” (C classification). The performance of classification and prediction by SVM were measured by 5-fold cross-validation. Datasets were randomly sampled and divided into 5 parts, 4 parts were used for training sets and the remainder one part for prediction. The performance of binary classification was assessed with sensitivity, specificity, accuracy and area under curve (AUC), where

Sensitivity = true positives/(true positives + false negatives)

Specificity = true negatives/(true negatives + false positives)

Accuracy = (true positives + true negatives)/(true positives + false positives + true negatives + false negatives)

Sensitivity, specificity, accuracy and AUC were computed using the cumulative results of repeating classifications 10 times. AUC was computed by an R package pROC [23]. The performance of multiclass classification was assessed by the accuracy computed from the fraction of correct predictions within total prediction number.

### 4.5. Hierarchical Clustering Dendrogram and Heatmaps

All of the GSR indices in each gene set for each subtype were averaged and underwent hierarchical clustering with the R function “heatmap” as default. This function would execute hierarchical clustering, and drew dendrogram and heatmaps.

### 4.6. Set Analysis

All possible logical relations among the deregulated gene sets of the four EOC subtype groups was evaluated by set analysis and displayed by Venn diagram using an R package “VennDiagram” (version 1.6.16, downloaded from the comprehensive R archive network (CRAN), https://cran.r-project.org/index.html).

### 4.7. Exploratory Factor Analysis for the Deregulated GO Terms and Establishment of GO Trees

The deregulated GO terms of *p* values <0.001 were selected for EFA. EFA was executed with the R package “psych” (version 1.5.8). The number of factors to be extracted was determined by the function “pa.parellel”. The setting of factoring method used in this study was “pa” and the correlation matrix rotation method was “promax”. The tree of the deregulated GO terms was constructed and visualized in Portable Network Graphics (PNG) format constructed by the “RamiGO” [24], an R package providing functions to interact with the AmiGO 2 web server and retrieves GO trees.

### 4.8. Detection of Differentially Expressed Genes in the Four Subtypes of EOC

To discover the DEGs for each of the four EOC subtypes, we carried out integrative analysis with the downloaded DNA microarray datasets. The gene expression levels of all samples in each dataset were transformed and rescaled to cumulative proportion values from 0 (lowest expression) to 1 (highest expression) with an R package “YuGene” (version 1.1.5) before integration. The DEGs were discovered using linear model computed with empirical Bayes analysis by the functions “lmFit” and “eBayes” provided by the R package “limna” (version 3.26.9).

## 5. Conclusions

Investigating the pathogenesis of diseases with the functionomes not only makes a clear distinction among the different subtypes, but also provides a comprehensive view of the deregulated functions in these diseases. Our study demonstrated two overlapped but distinguishable deregulated function patterns among the four EOC subtypes. The first pattern, observed in CCC, EC and MC, showed a relatively moderate deregulation of functions involving the PI3K-related functions and chromatin assembly. The second pattern, found in SC, showed more severely deregulated functions associated with the control of cell cycle. These findings were compatible with the type I and II classifications proposed by the dualistic model of ovarian carcinogenesis. This study provided solid evidences to support this classification and was the first integrative analysis demonstrating this model.

## Figures and Tables

**Table 1:** Sample numbers and means of the gene set regularity indices for each subtype. The table displayed the sample numbers, means and SDs of GSR indices for the four EOC subtypes and the normal ovarian tissue controls computed through the GO term gene sets. The 136 normal ovarian tissue sample gene expression profiles were utilized as the control group for the all of the four EOC subtypes.

EOC Subtype	Sample	Control	Total	Sample Mean (SD)	Control Mean (SD)	*p* Value *
Clear cell	85	136	221	0.7438 (0.1171)	0.7727 (0.1329)	<0.001
Endometrioid	90	136	226	0.7434 (0.1260)	0.7731 (0.1326)	<0.001
Mucinous	48	136	184	0.7174 (0.1531)	0.7724 (0.1334)	<0.001
Serous	1093	136	1229	0.6694 (0.1997)	0.7697 (0.1589)	<0.001

SD: standard deviation; GSR: gene set regularity; EOC: epithelial ovarian carcinoma; GO: Gene Ontology; * Mann Whitney *U* test.

**Table 2:** Accuracies of the binary and multiclass classification and prediction by machine learning. This table displayed the performances of the binary (each subtype vs. control group) and multiclass classification (among the four subtype groups) and prediction by SVM with the GSR indices computed through the GO terms. The sensitivities, specificities, AUC, accuracies and the SD were measured by five-fold cross-validation. Each measurement was computed by the cumulative 10 results of repeated classifications and predictions. SVM: support vector machine; GSR: gene set regularity; GO: Gene Ontology; AUC: area under curve; SD: standard deviation; NA: not available.

EOC Subtype	Sensitivity (SD)	Specificity (SD)	Accuracy (SD)	AUC
Clear Cell	1.0000 (0.0000)	1.0000 (0.0000)	1.0000 (0.0000)	1.0000
Endometrioid	0.9724 (0.0463)	1.0000 (0.0000)	0.9888 (0.0188)	0.9868
Mucinous	0.9582 (0.0559)	1.0000 (0.0000)	0.9818 (0.0139)	0.9805
Serous	0.9930 (0.0004)	0.9680 (0.0269)	0.9902 (0.0004)	0.9807
Multiclass	NA	NA	0.9555 (0.0112)	NA

**Table 3:** The top 15 deregulated Gene Ontology (GO) terms of the four subtype groups ranked by the *p* values. This table displayed the top 15 significantly deregulated GO terms of each subtype. GO: Gene Ontology.

Clear Cell	Endometrioid	Mucinous	Serous
Cofactor Transport	Cofactor Transporter Activity	Aldo Keto Reductase Activity	Protein Tyrosine Activity
Inositol or Phosphatidylinositol Phosphatase Activity	Secretin Like Receptor Activity	Secretin Like Receptor Activity	Oxidoreductase Activity Acting on The Aldehyde or OXO Group of Donors
Rho Guanyl Nucleotide Exchange Factor Activity	Carbohydrate Biosynthetic Process	Vitamin Transport	Homophilic Cell Adhesion
Small Conjugating Protein Binding	Regulation of Viral Reproduction	Rho Guanyl Nucleotide Exchange Factor Activity	Regulation of Actin Filament Length
Ubiquitin Binding	Calcium Independent Cell Adhesion	Small Conjugating Protein Binding	Regulation of Actin Polymerization and or Depolymerization
Regulation of Viral Reproduction	Coenzyme Binding	Ubiquitin Binding	Regulation of Cellular Component Size
Vitamin Transport	Sulfotransferase Activity	Calcium Channel Activity	Vitamin Metabolic Process
Steroid Hormone Receptor Binding	Inositol or Phosphatidylinositol Phosphatase Activity	Negative Regulation of Immune System Process	Spindle Pole
Histone Deacetylase Binding	Calcium Channel Activity	Carbohydrate Biosynthetic Process	Negative Regulation of Cellular Component Organization and Biogenesis
Oxidoreductase Activity Acting on the CH NH Group of Donors	Cofactor Binding	Inositol or Phosphatidylinositol Phosphatase Activity	Spindle
Transmembrane Receptor Protein Tyrosine Kinase Activity	Transferase Activity Transferring Sulfur Containing Groups	Neuropeptide Binding	Innate Immune Response
Protein Tyrosine Kinase Activity	Oxidoreductase Activity Acting on The Aldehyde or OXO Group of Donors	Neuropeptide Receptor Activity	Negative Regulation of Cell Proliferation
Insoluble Fraction	Vitamin Transport	Transmembrane Receptor Protein Tyrosine Kinase Activity	Regulation of Organelle Organization and Biogenesis
Carbohydrate Biosynthetic Process	Transmembrane Receptor Protein Tyrosine Kinase Activity	Innate Immune Response	Single Stranded DNA Binding
Ras Guanyl Nucleotide Exchange Factor Activity	Rho Guanyl Nucleotide Exchange Factor Activity	Cofactor Transporter Activity	Oxidoreductase Activity Acting on The Aldehyde or OXO Group of Donorsnad or Nadp As Acceptor

**Table 4:** The top 15 deregulated Reactome pathways ranked by the *p* values of each EOC subtype group. This table partially displayed the significantly deregulated Reactome pathways of each subtype. Only the top 15 deregulated Reactome pathway gene sets were listed.

Clear Cell	Endometrioid	Mucinous	Serous
Downregulation of ERBB2 ERBB3 Signaling	Downregulation of ERBB2 ERBB3 Signaling	Organic Cation Anion Zwitterion Transport	Ca Dependent Events
Negative Regulation of the PI3K AKT Network	CD28 Dependent PI3K AKT Signaling	Downregulation of ERBB2 ERBB3 Signaling	DARPP 32 Events
Activated AMPK Stimulates Fatty Acid Oxidation in Muscle	Organic Cation Anion Zwitterion Transport	Olfactory Signaling Pathway	Signaling by Robo Receptor
PERK Regulated Gene Expression	Nef Mediated Downregulation of MHC Class I Complex Cell Surface Expression	Digestion of Dietary Carbohydrate	Plc Beta Mediated Events
Ethanol Oxidation	GABA Synthesis Release Reuptake and Degradation	PI3K Events in ERBB2 Signaling	COPI Mediated Transport
Phospholipase C Mediated Cascade	Negative Regulation of the PI3K AKT Network	Regulation of Insulin Like Growth Factor Igf Activity by Insulin Like Growth Factor Binding Proteins Igfbps	Sphingolipid De Novo Biosynthesis
Regulation of Rheb Gtpase Activity By AMPK	Inhibition of The Proteolytic Activity of APC C Required for The Onset of Anaphase By Mitotic Spindle Checkpoint Components	Regulated Proteolysis of P75NTR	DAG and IP3 Signaling
PI3K Cascade	NCAM1 Interactions	Activated AMPK Stimulates Fatty Acid Oxidation in Muscle	NCAM1 Interactions
Beta Defensins	GPVI Mediated Activation Cascade	Nef Mediated Downregulation Of MHC Class I Complex Cell Surface Expression	G0 and Early G1
FGFR Ligand Binding and Activation	Phosphorylation of The APC C	Peptide Ligand Binding Receptors	Gα Z Signalling Events
Common Pathway	Termination of O Glycan Biosynthesis	Class A1 Rhodopsin Like Receptors	MHC Class II Antigen Presentation
Activation of Genes by ATF4	Regulation of Rheb Gtpase Activity by AMPK	Intrinsic Pathway	Signaling by PDGF
GPVI Mediated Activation Cascade	Activated Ampk Stimulates Fatty Acid Oxidation in Muscle	CD28 Dependent PI3K AKT Signaling	HS GAG Biosynthesis
PI3K Cascade	Conversion From APC C CDC20 to APC C Cdh1 in Late Anaphase	Endogenous Sterols	Chondroitin Sulfate Dermatan Sulfate Metabolism
Insulin Receptor Signalling Cascade	APC C CDC20 Mediated Degradation of Cyclin B	Formation of Fibrin Clot Clotting Cascade	Abacavir Transport and Metabolism

**Table 5:** The top 100 up- and down-regulated differentially expressed genes of the four EOC subtype groups. The genes were ranked by their *p* values.

	Clear Cell				Endometrioid				Mucinous				Serous			
	Down-Regulation		Up-Regulation		Down-Regulation		Up-Regulation		Down-Regulation		Up-Regulation		Down-Regulation		Up-Regulation	
Ranking	Gene	*p* Value	Gene	*p* Value	Gene	*p* Value	Gene	*p* Value	Gene	*p* Value	Gene	*p* Value	Gene	*p* Value	Gene	*p* Value
1	*EIF3F*	7.35 × 10^−109^	*TOMM7*	1.67 × 10^−118^	*EIF3F*	8.76 × 10^−114^	*TOMM7*	9.66 × 10^−107^	*EIF3F*	2.22 × 10^−101^	*RPL23*	7.94 × 10^−88^	*AOX1*	3.51 × 10^−133^	*C14orf2*	8.15 × 10^−78^
2	*RPL21*	9.70 × 10^−89^	*RPL24*	1.23 × 10^−109^	*RPS13*	9.13 × 10^−98^	*RPL34*	1.96 × 10^−96^	*TOMM7*	7.54 × 10^−94^	*PLS3*	6.95 × 10^−86^	*EIF3F*	2.00 × 10^−132^	*COX6B1*	2.59 × 10^−66^
3	*PRNP*	1.88 × 10^−81^	*RPS13*	9.31 × 10^−102^	*RPS11*	5.65 × 10^−95^	*RPL23*	3.91 × 10^−95^	*RPL34*	2.75 × 10^−89^	*FHL2*	5.95 × 10^−82^	*DFNA5*	1.26 × 10^−128^	*TRIAP1*	3.44 × 10^−65^
4	*RPL13*	4.78 × 10^−80^	*EIF3L*	1.52 × 10^−101^	*RPL27*	2.10 × 10^−93^	*ALDH9A1*	5.65 × 10^−95^	*RPS13*	1.60 × 10^−84^	*ALDH9A1*	6.07 × 10^−82^	*PTGIS*	6.85 × 10^−125^	*RBX1*	9.37 × 10^−63^
5	*CAV1*	3.04 × 10^−78^	*RPS11*	1.71 × 10^−98^	*DFNA5*	4.74 × 10^−92^	*PLS3*	1.30 × 10^−94^	*RPS11*	2.85 × 10^−83^	*RPS27L*	8.49 × 10^−80^	*TSPAN5*	7.08 × 10^−124^	*CGRRF1*	1.25 × 10^−61^
6	*DFNA5*	1.76 × 10^−76^	*ITM2B*	6.57 × 10^−98^	*RPL39*	1.64 × 10^−89^	*ITM2B*	6.36 × 10^−94^	*RPS15*	7.86 × 10^−82^	*SEC31A*	8.73 × 10^−80^	*BAMBI*	2.13 × 10^−108^	*LSM6*	6.16 × 10^−60^
7	*RPS28*	4.73 × 10^−74^	*RPL27*	7.35 × 10^−98^	*RPL41*	1.38 × 10^−86^	*RPS15*	3.67 × 10^−93^	*RPL27*	2.31 × 10^−80^	*RRAGA*	2.54 × 10^−78^	*SPOCK1*	2.13 × 10^−108^	*COX5A*	1.71 × 10^−59^
8	*CALD1*	3.06 × 10^−70^	*RPL17*	5.33 × 10^−97^	*SGK1*	1.71 × 10^−85^	*RPL36AL*	2.03 × 10^−90^	*DFNA5*	2.26 × 10^−79^	*YPEL5*	3.86 × 10^−78^	*GFPT2*	8.91 × 10^−107^	*TIMM8B*	1.54 × 10^−58^
9	*PMP22*	5.06 × 10^−69^	*RPS15*	2.73 × 10^−94^	*RPLP2*	6.38 × 10^−85^	*RPL32*	1.64 × 10^−89^	*RPL32*	1.61 × 10^−77^	*RPL36*	2.04 × 10^−77^	*C21orf62*	1.35 × 10^−106^	*SNX6*	1.62 × 10^−58^
10	*TPM1*	8.35 × 10^−69^	*RPL5*	2.97 × 10^−92^	*PRNP*	3.01 × 10^−84^	*LAPTM4A*	1.91 × 10^−88^	*RPL39*	1.61 × 10^−77^	*RPL36AL*	4.12 × 10^−77^	*FLRT2*	5.29 × 10^−104^	*IER3IP1*	1.88 × 10^−58^
11	*RPL10*	1.07 × 10^−67^	*PLS3*	1.17 × 10^−91^	*CAV1*	5.20 × 10^−84^	*SRP14*	5.89 × 10^−88^	*PRNP*	3.65 × 10^−77^	*LAPTM4A*	1.20 × 10^−76^	*NDN*	2.35 × 10^−103^	*MGST2*	2.04 × 10^−57^
12	*PTGIS*	1.60 × 10^−66^	*RPS3A*	1.42 × 10^−91^	*UROD*	1.72 × 10^−82^	*RPL36*	1.43 × 10^−87^	*SGK1*	5.08 × 10^−77^	*ANXA5*	4.42 × 10^−76^	*GPRASP1*	5.93 × 10^−103^	*METTL5*	2.38 × 10^−57^
13	*DCN*	1.88 × 10^−66^	*RPL39*	7.65 × 10^−91^	*RPS28*	3.11 × 10^−81^	*RPL6*	2.48 × 10^−86^	*RPL30*	2.92 × 10^−75^	*DSTN*	5.00 × 10^−74^	*IGFBP6*	3.90 × 10^−102^	*MRPS14*	3.94 × 10^−57^
14	*NDN*	1.02 × 10^−65^	*RPS27L*	7.68 × 10^−90^	*PMP22*	4.40 × 10^−81^	*RPL30*	2.59 × 10^−86^	*PMP22*	3.66 × 10^−74^	*OAT*	5.21 × 10^−74^	*RPS11*	6.71 × 10^−101^	*JMJD6*	1.32 × 10^−56^
15	*HNRNPA1L2*	3.57 × 10^−64^	*RPL23*	9.84 × 10^−90^	*TIMP2*	5.47 × 10^−81^	*GABARAP*	8.47 × 10^−86^	*RPL6*	7.61 × 10^−74^	*CD99*	2.58 × 10^−73^	*ZFPM2*	4.41 × 10^−96^	*NOP10*	1.41 × 10^−56^
16	*SH3BP4*	1.22 × 10^−63^	*RPL36AL*	1.60 × 10^−89^	*PTGIS*	1.43 × 10^−76^	*OAT*	7.37 × 10^−85^	*UROD*	2.92 × 10^−73^	*DPYSL2*	4.47 × 10^−73^	*RPS18*	7.65 × 10^−95^	*NFU1*	1.52 × 10^−56^
17	*RPS14*	1.31 × 10^−63^	*RPL34*	1.98 × 10^−89^	*NDN*	2.18 × 10^−76^	*LTA4H*	1.92 × 10^−84^	*RPLP2*	9.80 × 10^−72^	*CAMLG*	4.55 × 10^−73^	*ME1*	9.97 × 10^−94^	*PIGP*	1.81 × 10^−56^
18	*FHL1*	6.68 × 10^−63^	*ALDH9A1*	2.31 × 10^−89^	*GFPT2*	1.20 × 10^−73^	*FHL2*	2.74 × 10^−84^	*RPL41*	1.66 × 10^−71^	*GABARAPL2*	5.06 × 10^−73^	*RPL27A*	1.68 × 10^−93^	*ITGB3BP*	2.15 × 10^−55^
19	*HUWE1*	7.18 × 10^−63^	*RPL3*	6.82 × 10^−89^	*VCL*	2.20 × 10^−73^	*UBB*	2.83 × 10^−84^	*RPL15*	3.77 × 10^−70^	*FAU*	7.09 × 10^−73^	*SERPINE2*	5.58 × 10^−93^	*RNF139*	2.65 × 10^−55^
20	*SERPINE2*	9.91 × 10^−63^	*RPL36*	1.59 × 10^−88^	*AMIGO2*	1.07 × 10^−72^	*RRAGA*	6.11 × 10^−84^	*RPS28*	1.07 × 10^−69^	*SRP14*	1.13 × 10^−72^	*UROD*	4.27 × 10^−92^	*C19orf53*	5.82 × 10^−55^
21	*TACC1*	3.81 × 10^−62^	*RPL30*	2.93 × 10^−88^	*LXN*	4.59 × 10^−72^	*CD99*	1.95 × 10^−83^	*UBB*	2.99 × 10^−69^	*ST13*	3.83 × 10^−72^	*TRPC1*	5.36 × 10^−92^	*SEC22B*	1.08 × 10^−54^
22	*LXN*	7.86 × 10^−62^	*RPL6*	9.56 × 10^−88^	*MEIS2*	3.01 × 10^−71^	*RPS24*	3.55 × 10^−83^	*RPS27A*	8.25 × 10^−69^	*TCEAL4*	5.30 × 10^−72^	*AMIGO2*	7.52 × 10^−92^	*DDIT3*	2.08 × 10^−54^
23	*IL6ST*	1.08 × 10^−61^	*RPL32*	1.05 × 10^−86^	*CRIM1*	7.32 × 10^−70^	*ST13*	3.57 × 10^−83^	*RPL10A*	9.99 × 10^−69^	*HTRA1*	9.66 × 10^−72^	*ERH*	1.00 × 10^−91^	*NOSIP*	8.18 × 10^−54^
24	*ZFPM2*	6.36 × 10^−61^	*RPL31*	3.52 × 10^−86^	*TACC1*	1.50 × 10^−69^	*SEC31A*	5.38 × 10^−83^	*RPS27*	1.85 × 10^−68^	*NDUFA4*	9.80 × 10^−72^	*DAPK1*	2.40 × 10^−91^	*ELP4*	1.23 × 10^−53^
25	*VAPA*	5.06 × 10^−60^	*RPS16*	5.06 × 10^−86^	*ZFP36L1*	3.10 × 10^−69^	*DPYSL2*	1.33 × 10^−82^	*NDN*	2.75 × 10^−68^	*FTO*	1.31 × 10^−71^	*PMP22*	5.50 × 10^−90^	*ATP5G1*	1.33 × 10^−53^
26	*MEIS2*	9.44 × 10^−60^	*TPT1*	6.28 × 10^−86^	*SGCE*	1.02 × 10^−68^	*YPEL5*	2.76 × 10^−82^	*RPS18*	2.91 × 10^−68^	*RPS24*	1.71 × 10^−71^	*VCL*	1.15 × 10^−89^	*C14orf1*	5.69 × 10^−53^
27	*C1S*	3.56 × 10^−59^	*ACTG1*	8.08 × 10^−86^	*IGFBP6*	1.69 × 10^−68^	*FAU*	3.91 × 10^−82^	*ZFAND5*	1.68 × 10^−67^	*GABARAP*	3.74 × 10^−71^	*DIRAS3*	1.56 × 10^−89^	*SDC4*	4.24 × 10^−52^
28	*BAMBI*	6.54 × 10^−59^	*SNX3*	9.76 × 10^−86^	*ZFPM2*	2.99 × 10^−68^	*RPS27L*	7.11 × 10^−82^	*RPL27A*	1.83 × 10^−67^	*REEP5*	9.51 × 10^−71^	*PRKCDBP*	6.25 × 10^−89^	*PDCD10*	8.22 × 10^−52^
29	*CDH11*	8.97 × 10^−59^	*CCNI*	1.18 × 10^−85^	*SERPINE2*	6.46 × 10^−68^	*CAMLG*	2.19 × 10^−81^	*AMIGO2*	3.98 × 10^−66^	*GNB2L1*	3.48 × 10^−69^	*PDGFD*	1.10 × 10^−88^	*CCDC25*	1.87 × 10^−51^
30	*PDGFRA*	4.98 × 10^−58^	*RPL13A*	4.88 × 10^−85^	*GSTM3*	1.76 × 10^−67^	*RPL10A*	5.37 × 10^−81^	*PTGIS*	1.35 × 10^−64^	*LTA4H*	3.48 × 10^−69^	*CLIP4*	1.39 × 10^−88^	*NOC3L*	3.10 × 10^−51^
31	*CYBRD1*	1.07 × 10^−57^	*RPS20*	2.26 × 10^−84^	*PDGFRA*	7.06 × 10^−67^	*DSTN*	1.02 × 10^−80^	*C1S*	1.02 × 10^−63^	*ERH*	5.04 × 10^−69^	*RPL23*	1.39 × 10^−88^	*SDHD*	4.27 × 10^−51^
32	*IGFBP6*	2.72 × 10^−57^	*BTF3*	2.72 × 10^−84^	*PLSCR4*	7.22 × 10^−67^	*RPL15*	1.02 × 10^−80^	*GFPT2*	4.97 × 10^−63^	*TMSB4X*	4.82 × 10^−68^	*PLS3*	2.25 × 10^−88^	*FAM96B*	4.47 × 10^−51^
33	*ZMIZ1*	3.24 × 10^−57^	*COX7C*	4.34 × 10^−84^	*CYBRD1*	1.44 × 10^−66^	*RPS18*	1.82 × 10^−80^	*VCL*	5.06 × 10^−63^	*HNRNPK*	4.82 × 10^−68^	*PAPSS2*	5.82 × 10^−88^	*DCTPP1*	8.65 × 10^−51^
34	*7−Sep*	3.73 × 10^−57^	*RPS12*	5.08 × 10^−84^	*ARMCX1*	3.81 × 10^−66^	*GABARAPL2*	1.88 × 10^−80^	*SGCE*	5.98 × 10^−63^	*CRTAP*	1.22 × 10^−67^	*ST3GAL5*	1.39 × 10^−87^	*MRPS35*	1.26 × 10^−50^
35	*PLSCR4*	1.04 × 10^−56^	*SRP14*	6.84 × 10^−84^	*DAPK1*	9.42 × 10^−66^	*ANXA5*	3.65 × 10^−80^	*ATP5A1*	3.58 × 10^−62^	*PALLD*	1.75 × 10^−67^	*CAMLG*	8.01 × 10^−86^	*PPP1CB*	1.46 × 10^−50^
36	*CAPN2*	2.02 × 10^−56^	*RPL41*	1.45 × 10^−83^	*ZCCHC24*	1.10 × 10^−65^	*ERH*	4.10 × 10^−80^	*ZFP36L1*	5.62 × 10^−62^	*TMSB10*	1.88 × 10^−67^	*CALB2*	2.38 × 10^−85^	*ATIC*	1.88 × 10^−50^
37	*FLRT2*	5.76 × 10^−56^	*CAMLG*	1.84 × 10^−83^	*AOX1*	1.15 × 10^−65^	*TCEAL4*	4.44 × 10^−80^	*BNIP3*	1.95 × 10^−61^	*LEPROT*	1.74 × 10^−66^	*HOXC6*	6.53 × 10^−85^	*MRPS33*	3.69 × 10^−50^
38	*GFPT2*	8.87 × 10^−56^	*FAU*	2.07 × 10^−83^	*DDR2*	2.29 × 10^−65^	*HTRA1*	5.70 × 10^−80^	*BAMBI*	4.34 × 10^−61^	*MORF4L1*	2.29 × 10^−66^	*NT5E*	1.07 × 10^−84^	*RAB32*	4.09 × 10^−50^
39	*DDR2*	1.19 × 10^−55^	*ATP5L*	2.53 × 10^−83^	*IFFO1*	4.57 × 10^−65^	*TMSB4X*	1.69 × 10^−79^	*SERPINE2*	2.87 × 10^−60^	*ADH5*	2.72 × 10^−66^	*LXN*	3.09 × 10^−84^	*MYL6B*	4.23 × 10^−50^
40	*RGL1*	1.89 × 10^−55^	*RPS4X*	6.11 × 10^−83^	*FLRT2*	5.39 × 10^−65^	*REEP5*	1.99 × 10^−79^	*TUBA1A*	4.49 × 10^−60^	*UBA52*	4.35 × 10^−66^	*GALC*	4.12 × 10^−84^	*EIF2S1*	4.48 × 10^−50^
41	*DAB2*	4.93 × 10^−55^	*RPSA*	9.43 × 10^−83^	*PAPSS2*	1.43 × 10^−64^	*RPS27*	2.52 × 10^−79^	*RGL1*	2.40 × 10^−59^	*PNRC2*	4.35 × 10^−66^	*SGK1*	4.67 × 10^−84^	*SGCB*	5.45 × 10^−50^
42	*NR3C1*	7.53 × 10^−55^	*GNB2L1*	9.98 × 10^−83^	*PRKCDBP*	1.59 × 10^−64^	*UBA52*	3.26 × 10^−79^	*CCT8*	4.12 × 10^−59^	*EID1*	5.10 × 10^−66^	*ALDH1A3*	7.40 × 10^−84^	*SNAPC5*	5.62 × 10^−50^
43	*ZCCHC24*	7.58 × 10^−55^	*ATP6V0E1*	1.35 × 10^−82^	*PROS1*	1.62 × 10^−64^	*NPTN*	3.81 × 10^−79^	*CYBRD1*	7.82 × 10^−59^	*NPTN*	5.13 × 10^−66^	*PLSCR4*	9.27 × 10^−84^	*ZZZ3*	1.37 × 10^−49^
44	*PROS1*	1.57 × 10^−54^	*RPL18*	1.91 × 10^−82^	*FZD7*	1.87 × 10^−64^	*RPL27A*	1.30 × 10^−78^	*ZFPM2*	1.02 × 10^−58^	*RPS26*	6.74 × 10^−66^	*VGLL3*	2.60 × 10^−83^	*PSMB3*	1.99 × 10^−49^
45	*FSTL1*	2.49 × 10^−54^	*RPS24*	3.78 × 10^−82^	*IGFBP5*	8.03 × 10^−64^	*TMSB10*	2.07 × 10^−78^	*PLSCR4*	1.89 × 10^−58^	*SLC25A3*	1.77 × 10^−65^	*COX7A2*	2.94 × 10^−83^	*CISD1*	2.63 × 10^−49^
46	*MYLK*	2.80 × 10^−54^	*EEF1G*	1.88 × 10^−81^	*RGS2*	1.71 × 10^−63^	*SLC25A3*	2.80 × 10^−78^	*DYRK1A*	4.52 × 10^−58^	*EIF3E*	1.81 × 10^−65^	*ALDH9A1*	5.65 × 10^−83^	*RTN3*	2.71 × 10^−49^
47	*ARMCX1*	3.45 × 10^−54^	*RRAGA*	6.51 × 10^−81^	*TSPAN5*	1.73 × 10^−63^	*HNRNPK*	7.96 × 10^−78^	*FZD7*	5.61 × 10^−58^	*TAX1BP3*	5.34 × 10^−65^	*FHL2*	6.12 × 10^−82^	*TMED3*	3.49 × 10^−49^
48	*FZD7*	4.01 × 10^−54^	*RPL35A*	1.11 × 10^−80^	*BAMBI*	1.81 × 10^−63^	*GNB2L1*	9.58 × 10^−78^	*CAPN2*	6.35 × 10^−58^	*LDHA*	6.73 × 10^−65^	*CYBRD1*	7.23 × 10^−82^	*CCDC59*	6.01 × 10^−49^
49	*IGFBP5*	6.73 × 10^−54^	*RPS17*	2.28 × 10^−80^	*CLIP4*	1.15 × 10^−62^	*MYL6*	1.30 × 10^−77^	*FLRT2*	7.64 × 10^−58^	*MYL6*	7.78 × 10^−65^	*SEMA3C*	1.20 × 10^−81^	*POLR2L*	6.23 × 10^−49^
50	*GAS1*	9.23 × 10^−54^	*CIRBP*	2.72 × 10^−80^	*HOXC6*	3.29 × 10^−62^	*FTO*	1.54 × 10^−77^	*IGFBP6*	1.30 × 10^−57^	*HSP90AA1*	8.80 × 10^−65^	*ATP10D*	3.70 × 10^−81^	*FAM53C*	8.50 × 10^−49^
51	*SEMA3C*	9.50 × 10^−54^	*TMSB4X*	3.69 × 10^−80^	*TGFB1I1*	3.77 × 10^−62^	*RPS26*	1.74 × 10^−77^	*ARMCX1*	2.18 × 10^−57^	*KLHDC2*	9.40 × 10^−65^	*DPYSL2*	3.70 × 10^−81^	*GOLPH3L*	9.76 × 10^−49^
52	*TXNRD1*	1.12 × 10^−53^	*RPL10A*	6.66 × 10^−80^	*OPTN*	4.04 × 10^−62^	*NDUFA4*	3.72 × 10^−77^	*TSPAN5*	2.64 × 10^−57^	*ISCU*	1.04 × 10^−64^	*FOXO1*	4.60 × 10^−81^	*NDUFA13*	1.14 × 10^−48^
53	*RNASE4*	1.60 × 10^−53^	*FHL2*	7.39 × 10^−80^	*APPBP2*	8.30 × 10^−62^	*HSP90AA1*	3.93 × 10^−77^	*SDC2*	2.86 × 10^−57^	*PDLIM1*	2.09 × 10^−64^	*DSTN*	8.10 × 10^−81^	*DUSP22*	1.27 × 10^−48^
54	*TSPAN5*	1.76 × 10^−53^	*ANXA5*	1.04 × 10^−79^	*ST3GAL5*	1.31 × 10^−61^	*EIF3E*	5.34 × 10^−77^	*SEMA3C*	3.16 × 10^−57^	*SPCS1*	2.52 × 10^−64^	*TIMP2*	2.78 × 10^−80^	*BET1*	1.32 × 10^−48^
55	*CFH*	2.76 × 10^−53^	*NDUFA4*	2.66 × 10^−79^	*CLDN11*	1.79 × 10^−61^	*ADH5*	7.61 × 10^−77^	*DDR2*	5.10 × 10^−57^	*SPARC*	4.80 × 10^−64^	*ANXA5*	3.30 × 10^−80^	*SEH1L*	1.35 × 10^−48^
56	*ALCAM*	5.43 × 10^−53^	*SGK1*	3.86 × 10^−79^	*FBN1*	5.06 × 10^−61^	*SPCS1*	9.99 × 10^−77^	*ZCCHC24*	6.92 × 10^−57^	*LXN*	5.79 × 10^−64^	*DNAJB9*	8.77 × 10^−80^	*AMD1*	1.46 × 10^−48^
57	*PRKCDBP*	1.19 × 10^−52^	*EEF1A1*	4.61 × 10^−79^	*TCEAL2*	1.17 × 10^−60^	*MORF4L1*	1.87 × 10^−76^	*IGFBP5*	9.53 × 10^−57^	*ATF4*	7.07 × 10^−64^	*GHR*	9.71 × 10^−80^	*RALB*	1.66 × 10^−48^
58	*CLIP4*	2.90 × 10^−52^	*RPS27*	6.26 × 10^−79^	*HEG1*	2.94 × 10^−60^	*MTCH1*	2.18 × 10^−76^	*PRKCDBP*	3.43 × 10^−56^	*UXT*	7.96 × 10^−64^	*HTRA1*	1.43 × 10^−79^	*PLEKHA1*	2.10 × 10^−48^
59	*ANTXR1*	3.16 × 10^−52^	*OAT*	1.18 × 10^−78^	*RBPMS*	6.53 × 10^−60^	*RPS27A*	2.98 × 10^−76^	*IFFO1*	1.12 × 10^−55^	*SEPW1*	8.64 × 10^−64^	*SDC2*	1.81 × 10^−79^	*KIAA1598*	2.21 × 10^−48^
60	*GALC*	3.85 × 10^−52^	*YPEL5*	2.06 × 10^−78^	*AKT3*	7.43 × 10^−60^	*SEC11A*	3.00 × 10^−76^	*SPOCK1*	2.09 × 10^−55^	*COX7A2*	1.60 × 10^−63^	*COX6C*	2.02 × 10^−79^	*GGCT*	2.51 × 10^−48^
61	*EMP3*	4.13 × 10^−52^	*FTL*	2.31 × 10^−78^	*SPOCK1*	9.65 × 10^−60^	*LDHA*	3.43 × 10^−76^	*CFH*	3.82 × 10^−55^	*BTG1*	1.61 × 10^−63^	*FTO*	2.75 × 10^−79^	*MAGOH*	3.31 × 10^−48^
62	*IFFO1*	6.17 × 10^−52^	*RPS6*	2.52 × 10^−78^	*CFH*	1.72 × 10^−59^	*FTH1*	7.91 × 10^−76^	*STAT2*	7.79 × 10^−55^	*PGM1*	1.68 × 10^−63^	*NDUFA1*	3.17 × 10^−79^	*TBPL1*	3.54 × 10^−48^
63	*HOXC6*	8.97 × 10^−52^	*RPS18*	1.35 × 10^−77^	*RAB8B*	7.02 × 10^−59^	*ISCU*	1.02 × 10^−75^	*CLIC4*	9.47 × 10^−55^	*COX6C*	2.69 × 10^−63^	*IKBKAP*	3.31 × 10^−79^	*TSPAN31*	3.79 × 10^−48^
64	*SPOCK1*	1.39 × 10^−51^	*RPL27A*	1.61 × 10^−77^	*BNC2*	1.21 × 10^−58^	*EID1*	1.46 × 10^−75^	*ANTXR1*	1.22 × 10^−54^	*UQCRQ*	3.22 × 10^−63^	*LRRC49*	4.20 × 10^−79^	*BTN3A2*	5.37 × 10^−48^
65	*AOX1*	2.07 × 10^−51^	*RPS27A*	1.68 × 10^−77^	*GLT8D2*	1.95 × 10^−58^	*TAX1BP3*	1.69 × 10^−75^	*GALC*	1.43 × 10^−54^	*FTH1*	3.25 × 10^−63^	*TCF21*	8.18 × 10^−79^	*MEA1*	6.93 × 10^−48^
66	*B3GNT1*	2.28 × 10^−51^	*UBC*	1.68 × 10^−77^	*PDGFD*	2.76 × 10^−58^	*COX7A2*	3.20 × 10^−75^	*ZMIZ1*	1.55 × 10^−54^	*NPC2*	3.31 × 10^−63^	*AFF1*	9.76 × 10^−79^	*NUP37*	8.14 × 10^−48^
67	*RGS2*	2.28 × 10^−51^	*GABARAPL2*	1.90 × 10^−77^	*EMP3*	4.25 × 10^−58^	*CCNG1*	4.06 × 10^−75^	*SERPING1*	2.10 × 10^−54^	*DYNLL1*	4.32 × 10^−63^	*FSTL1*	1.62 × 10^−78^	*NXN*	1.07 × 10^−47^
68	*BNC2*	3.57 × 10^−51^	*LTA4H*	2.69 × 10^−77^	*MYLK*	1.01 × 10^−57^	*ATF4*	4.81 × 10^−75^	*PLSCR3*	3.49 × 10^−54^	*RWDD1*	5.98 × 10^−63^	*ADH5*	2.40 × 10^−78^	*ADNP2*	1.08 × 10^−47^
69	*ST3GAL5*	8.15 × 10^−51^	*C6orf48*	3.35 × 10^−77^	*TRPC1*	1.35 × 10^−57^	*PGAM1*	9.94 × 10^−75^	*CLIP4*	4.46 × 10^−54^	*YWHAQ*	5.98 × 10^−63^	*RPL36*	4.09 × 10^−78^	*EDEM1*	1.39 × 10^−47^
70	*AHNAK*	9.52 × 10^−51^	*EIF3E*	3.47 × 10^−77^	*OLFML1*	1.86 × 10^−57^	*PARK7*	1.36 × 10^−74^	*CLDN11*	5.46 × 10^−54^	*SKP1*	8.57 × 10^−63^	*GBE1*	7.94 × 10^−78^	*S100A6*	1.66 × 10^−47^
71	*TIPARP*	9.98 × 10^−51^	*ST13*	1.04 × 10^−76^	*COL16A1*	2.92 × 10^−57^	*PGM1*	1.91 × 10^−74^	*FYCO1*	6.09 × 10^−54^	*CTNNAL1*	9.54 × 10^−63^	*CUL3*	8.09 × 10^−78^	*FIS1*	1.69 × 10^−47^
72	*FBN1*	3.72 × 10^−50^	*ESD*	1.25 × 10^−76^	*ATP10D*	6.19 × 10^−57^	*LEPROT*	4.87 × 10^−74^	*MYLK*	1.86 × 10^−53^	*LAMP1*	1.09 × 10^−62^	*FGF2*	2.12 × 10^−77^	*RAB11FIP2*	2.00 × 10^−47^
73	*TCEAL2*	5.69 × 10^−50^	*UBA52*	2.38 × 10^−76^	*MAGEH1*	6.93 × 10^−57^	*NPC2*	6.09 × 10^−74^	*RNF38*	2.24 × 10^−53^	*IMPDH2*	1.30 × 10^−62^	*RRAGA*	2.76 × 10^−77^	*PPP1R8*	2.10 × 10^−47^
74	*SEPP1*	1.54 × 10^−49^	*MYL6*	2.51 × 10^−76^	*NAP1L3*	8.78 × 10^−57^	*RAC1*	6.70 × 10^−74^	*ST3GAL5*	3.61 × 10^−53^	*STX12*	1.31 × 10^−62^	*REEP1*	3.46 × 10^−77^	*NIPA2*	2.37 × 10^−47^
75	*TCF7L2*	2.39 × 10^−49^	*TIMP2*	3.11 × 10^−76^	*CAV2*	5.95 × 10^−56^	*PALLD*	8.77 × 10^−74^	*TGFB1I1*	4.12 × 10^−53^	*NDUFA1*	1.37 × 10^−62^	*HAS1*	5.77 × 10^−77^	*PNPO*	2.38 × 10^−47^
76	*AKT3*	2.56 × 10^−49^	*UBB*	4.48 × 10^−76^	*PDGFRL*	5.01 × 10^−55^	*PNRC2*	9.95 × 10^−74^	*TCEAL2*	7.67 × 10^−53^	*RNF11*	1.40 × 10^−62^	*RPL37*	1.03 × 10^−76^	*UBE2L6*	2.77 × 10^−47^
77	*CLDN11*	2.84 × 10^−49^	*COX6C*	5.01 × 10^−76^	*TGFBR2*	5.55 × 10^−55^	*SKP1*	1.64 × 10^−73^	*RBPMS*	7.72 × 10^−53^	*SEC11A*	1.45 × 10^−62^	*JAM3*	1.16 × 10^−76^	*ENY2*	3.05 × 10^−47^
78	*NFIB*	2.88 × 10^−49^	*TMSB10*	7.86 × 10^−76^	*GPR137B*	6.65 × 10^−55^	*COX6C*	2.24 × 10^−73^	*SULF1*	8.83 × 10^−53^	*LSM14A*	1.75 × 10^−62^	*RGL1*	3.20 × 10^−76^	*RBMX2*	3.26 × 10^−47^
79	*PDGFD*	3.10 × 10^−49^	*UROD*	7.86 × 10^−76^	*SULF1*	7.43 × 10^−55^	*TM2D3*	2.24 × 10^−73^	*AOX1*	1.82 × 10^−52^	*SCARB2*	1.89 × 10^−62^	*KLF2*	4.96 × 10^−76^	*NME4*	4.03 × 10^−47^
80	*RAB8B*	7.57 × 10^−49^	*PCNP*	1.79 × 10^−75^	*G0S2*	7.82 × 10^−55^	*PSAP*	2.57 × 10^−73^	*FOXJ3*	2.84 × 10^−52^	*TERF2IP*	1.89 × 10^−62^	*LDHA*	5.59 × 10^−76^	*TSN*	5.05 × 10^−47^
81	*HEG1*	9.15 × 10^−49^	*DSTN*	3.82 × 10^−75^	*ALDH1A3*	8.41 × 10^−55^	*NDUFA1*	2.75 × 10^−73^	*ZNF532*	3.61 × 10^−52^	*CRIM1*	3.23 × 10^−62^	*RAP1B*	6.45 × 10^−76^	*KPNA6*	7.18 × 10^−47^
82	*MAGEH1*	4.66 × 10^−48^	*RPL23A*	4.49 × 10^−75^	*PROCR*	1.25 × 10^−54^	*DYNLL1*	2.83 × 10^−73^	*FBN1*	4.29 × 10^−52^	*RASA1*	3.64 × 10^−62^	*VLDLR*	1.40 × 10^−75^	*COMMD8*	8.03 × 10^−47^
83	*GLT8D2*	4.95 × 10^−48^	*HSPA8*	1.46 × 10^−74^	*ANTXR1*	3.39 × 10^−54^	*CYB5R3*	3.30 × 10^−73^	*HEG1*	1.58 × 10^−51^	*LEPROTL1*	4.46 × 10^−62^	*TFPI*	2.30 × 10^−75^	*ASH1L*	8.67 × 10^−47^
84	*NT5E*	5.68 × 10^−48^	*HSP90AA1*	1.76 × 10^−74^	*ALDH1A2*	3.79 × 10^−54^	*KLHDC2*	5.79 × 10^−73^	*TNS3*	1.99 × 10^−51^	*TCF25*	4.54 × 10^−62^	*EHBP1*	2.43 × 10^−75^	*PEX11B*	8.82 × 10^−47^
85	*MAST4*	1.08 × 10^−47^	*ANP32B*	1.97 × 10^−74^	*PRKAR2B*	4.07 × 10^−54^	*LAMP1*	6.47 × 10^−73^	*BNC2*	3.07 × 10^−51^	*CCNG1*	4.73 × 10^−62^	*GPR176*	2.50 × 10^−75^	*MKKS*	1.13 × 10^−46^
86	*PTPRO*	1.13 × 10^−47^	*HINT1*	3.85 × 10^−74^	*CBX7*	4.38 × 10^−54^	*MXI1*	7.22 × 10^−73^	*MAP4*	3.93 × 10^−51^	*MXI1*	5.81 × 10^−62^	*RPS26*	3.55 × 10^−75^	*DUSP11*	1.22 × 10^−46^
87	*ZBED5*	3.98 × 10^−47^	*YWHAQ*	5.81 × 10^−74^	*MCC*	4.49 × 10^−54^	*ATP5J*	1.12 × 10^−72^	*AKT3*	4.95 × 10^−51^	*PSAP*	8.88 × 10^−62^	*CAPN2*	3.59 × 10^−75^	*ZMYND11*	1.31 × 10^−46^
88	*KLF2*	6.13 × 10^−47^	*EIF1*	7.34 × 10^−74^	*BEX1*	1.01 × 10^−53^	*RPL35*	1.27 × 10^−72^	*ROBO1*	6.39 × 10^−51^	*RPL35*	9.78 × 10^−62^	*EMP3*	4.30 × 10^−75^	*GCSH*	1.39 × 10^−46^
89	*OLFML1*	7.40 × 10^−47^	*RAC1*	1.06 × 10^−73^	*GPRASP1*	1.21 × 10^−53^	*YWHAQ*	1.76 × 10^−72^	*ARL3*	7.05 × 10^−51^	*MTCH1*	1.06 × 10^−61^	*SEC11A*	4.40 × 10^−75^	*MED7*	1.41 × 10^−46^
90	*RGS4*	9.61 × 10^−47^	*RPS26*	1.22 × 10^−73^	*RGS4*	1.41 × 10^−53^	*UQCRQ*	1.94 × 10^−72^	*CTSK*	1.11 × 10^−50^	*RPL8*	1.08 × 10^−61^	*MSRB2*	4.95 × 10^−75^	*C1orf54*	1.48 × 10^−46^
91	*ROBO1*	1.00 × 10^−46^	*SLC25A3*	1.33 × 10^−73^	*TBL1X*	1.52 × 10^−53^	*NEK7*	2.39 × 10^−72^	*MYH10*	1.67 × 10^−50^	*C14orf2*	1.29 × 10^−61^	*YPEL5*	4.98 × 10^−75^	*TSPO*	1.55 × 10^−46^
92	*BEX1*	2.45 × 10^−46^	*TCEAL4*	1.35 × 10^−73^	*STAT2*	2.18 × 10^−53^	*RPL37*	2.57 × 10^−72^	*EMP3*	1.94 × 10^−50^	*ARF4*	1.34 × 10^−61^	*MCC*	7.04 × 10^−75^	*ACVR2A*	1.62 × 10^−46^
93	*SULF1*	2.81 × 10^−46^	*RPS2*	1.76 × 10^−73^	*IKBKAP*	2.43 × 10^−53^	*CTNNAL1*	3.40 × 10^−72^	*MAGEH1*	2.11 × 10^−50^	*SH3BGRL*	1.58 × 10^−61^	*CAV2*	9.72 × 10^−75^	*GRSF1*	1.96 × 10^−46^
94	*FBXL7*	2.96 × 10^−46^	*RPLP2*	1.77 × 10^−73^	*NT5E*	6.01 × 10^−53^	*PTGES3*	8.90 × 10^−72^	*SALL2*	2.54 × 10^−50^	*MEIS2*	1.60 × 10^−61^	*TBC1D4*	9.72 × 10^−75^	*POLR2H*	2.27 × 10^−46^
95	*PROCR*	8.86 × 10^−46^	*DPYSL2*	2.09 × 10^−73^	*PSD3*	6.66 × 10^−53^	*UXT*	9.38 × 10^−72^	*RHOQ*	2.56 × 10^−50^	*AFF1*	1.88 × 10^−61^	*SLC25A3*	1.19 × 10^−74^	*THYN1*	2.31 × 10^−46^
96	*ABCA8*	9.09 × 10^−46^	*REEP5*	2.23 × 10^−73^	*PTRF*	8.14 × 10^−53^	*LSM14A*	9.94 × 10^−72^	*BEX1*	5.32 × 10^−50^	*PNMA1*	2.20 × 10^−61^	*LEPROTL1*	1.42 × 10^−74^	*UBE2V2*	3.01 × 10^−46^
97	*SALL2*	9.31 × 10^−46^	*MTCH1*	2.59 × 10^−73^	*PMM1*	1.29 × 10^−52^	*RPL8*	1.09 × 10^−71^	*SMARCA1*	6.06 × 10^−50^	*VAMP3*	2.62 × 10^−61^	*APPBP2*	1.54 × 10^−74^	*HMGCL*	3.30 × 10^−46^
98	*NAP1L3*	9.44 × 10^−46^	*LEPROT*	3.68 × 10^−73^	*GNAI1*	7.00 × 10^−52^	*ZFAND5*	1.17 × 10^−71^	*LDOC1*	1.94 × 10^−49^	*RPL37*	3.27 × 10^−61^	*MAPRE2*	1.69 × 10^−74^	*CSRP2*	3.34 × 10^−46^
99	*DKK3*	1.89 × 10^−45^	*UQCRQ*	4.99 × 10^−73^	*LAMA4*	8.23 × 10^−52^	*NCOA4*	1.33 × 10^−71^	*PDGFD*	2.13 × 10^−49^	*TIMP1*	3.27 × 10^−61^	*SPCS1*	2.22 × 10^−74^	*GPN3*	3.68 × 10^−46^
100	*ANXA6*	2.16 × 10^−45^	*SEC31A*	5.53 × 10^−73^	*ATP8B2*	1.94 × 10^−51^	*C14orf2*	1.58 × 10^−71^	*CTSF*	2.31 × 10^−49^	*PTGES3*	5.14 × 10^−61^	*TUBA1A*	3.27 × 10^−74^	*YIPF1*	3.70 × 10^−46^

## Supplementary Materials

The following are available online at www.mdpi.com/1422-0067/17/8/1272/s1.

## Abbreviations

EOC	Epithelial Ovarian Carcinoma
CCC	Clear Cell Carcinoma
EC	Endometrioid Carcinoma
MC	Mucinous Carcinoma
SC	Serous Carcinoma
GSR	Gene Set Regularity
DEG	Differentially Expressed Gene
DAG	Directed Acyclic Graph
GO	Gene Ontology
MSigDB	Molecular Signatures Database
EFA	Exploratory Factor Analysis
NCBI	National Center for Biotechnology Information
GEO	Gene Expression Omnibus
SD	Standard Deviation
SVM	Support Vector Machine
AUC	Area under Curve
GPCR	G Protein Coupled Receptor
HGNC	HUGO Gene Nomenclature Committee
DIRAC	Differential Rank Conservation,
RTK	Receptor Tyrosine Kinases
EGFR	Epidermal Growth Factor Receptor
CRAN	Comprehensive R Archive Network
