# Supplementary material for: Gene Set−Based Integrative Analysis Revealing Two Distinct Functional Regulation Patterns in Four Common Subtypes of Epithelial Ovarian Cancer
Source: Int J Mol Sci. 2016 Aug 5;17(8):1272. doi: 10.3390/ijms17081272 (PMC5000670; doi:10.3390/ijms17081272)
Supplement: Supplementary file 1 [file ijms-17-01272-s001.zip › ijms-139371-Supplementary Materials/ijms-139371-suplementary Figures.pdf]

Supplementary Materials: Gene Set-Based Integrative Analysis Revealing Two Distinct Functional Regulation Patterns in Four Common Subtypes of Epithelial Ovarian Cancer

Chia-Ming Chang, Chi-Mu Chuang, Mong-Lien Wang, Yi-Ping Yang, Jen-Hua Chuang, Ming-Jie Yang, Ming-Shyen Yen, Shih-Hwa Chiou and Cheng-Chang Chang

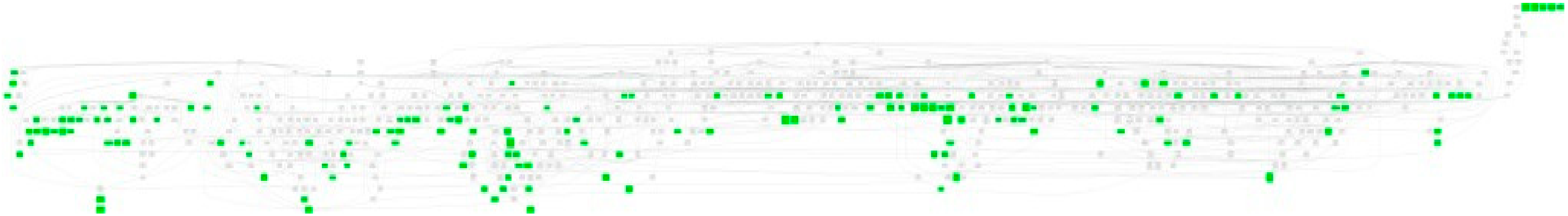

Figure S1. Tree of deregulated GO terms for clear cell carcinoma.

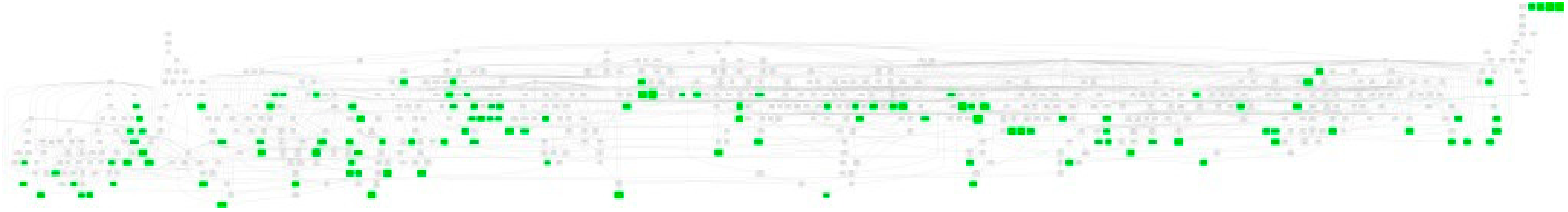

Figure S2. Tree of deregulated GO terms for endometrioid carcinoma.

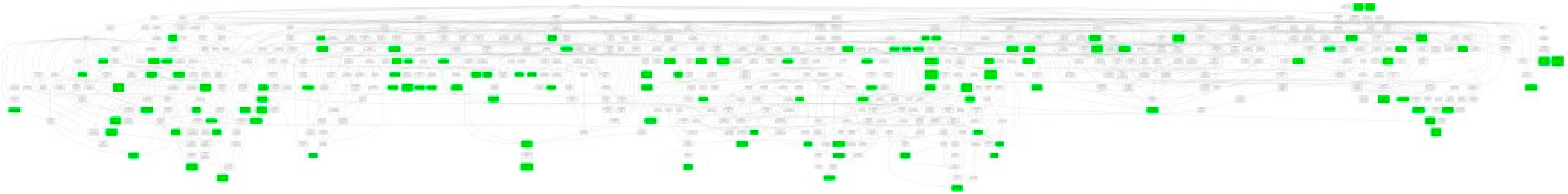

Figure S3. Tree of deregulated GO terms for mucinous carcinoma.

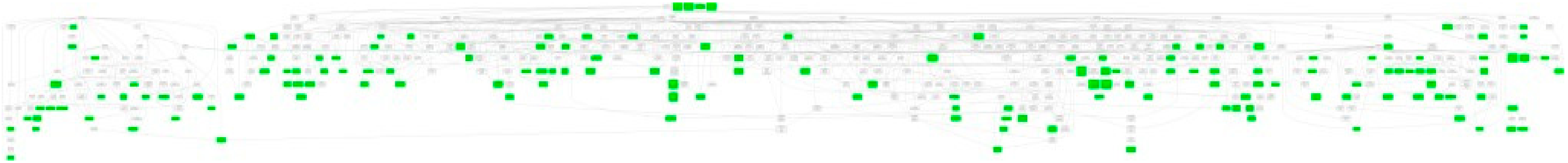

Figure S4. Tree of deregulated GO terms for serous carcinoma.
